# Supplementary material for: Learning curve and surgical outcome of robotic assisted colorectal surgery with ERAS program
Source: Sci Rep. 2022 Nov 29;12:20566. doi: 10.1038/s41598-022-24665-w (PMC9709162; doi:10.1038/s41598-022-24665-w)
Supplement: Supplementary file 2 — Supplementary Information 2. [file 41598_2022_24665_MOESM2_ESM.zip › RAL Raw data-Ñ[▒K/069/HS_ñw╝╨░O▒Kñσ.pdf]

正式摘要(電子簽章：000000213950998)		
W55 / W55 ☐英文標題(注意:無英文姓名-請補病人基本檔!)	☐【讀取】憑證	

臺中榮民總醫院
出院病歷摘要

轉入號 : 轉出號 :

[redacted]

(8)流水編號	
(9)入院日期	110/03/30 (2021) 直腸外科 W55 - 251
(10)轉科日期	
(11)出院日期	110/04/07 (2021) 住院天數計 8 日

診斷

- 入院 :1. Squamous cell carcinoma of low rectum
- 出院 :1. SCC-PD, with basaloid feature of low rectum, cT3N0M0
  s/p CCRT with cisplatin(40/m2) on 2020/9/8, 9/14, 9/25, 10/5, 10/12, 11/06
  with persistent disease
  s/p robotic-assisted APR on 2021/3/31
  2. OP history of thyroid tumor excision

主訴

- For scheduled robotic APR.

病史

- This 64 year-old female had history of
  1. SCC-PD, with basaloid feature of low rectum, cT3N0M0
  s/p CCRT with cisplatin(40/m2) on 2020/9/8, 9/14, 9/25, 10/5, 10/12, 11/06
  2. OP history of thyroid tumor excision
  She suffered from anal swelling without pain, a nodule at anus was noticed by the patient, combined with small caliber of stool in 2019.12. Sigmoid colon scope showed tumor at 2 cm FAV, s/p biopsy revealed squamous cell carcinoma, poorly differentiated, with basaloid feature. She accepted neoadjuvant CCRT first since 2020/9. After finishing neoadjuvant course, MRI pelvis showed anal cancer post neoadjuvant CCRT, with regression change of tumor size as compare with previous MR on 20201205. Lung CT revealed no obvious meatastasis lesion. SCC: 1.2 ng/ml. After discussing surgery option with patient, she decided robotic APR. Under the impression of SCC-PD, with basaloid feature of low rectum, cT3N0M0, she was admitted for surgical intervention and further treatment.

體檢發現

- Anal inspection:palpable mass on 3"
  10/20 hemorrhoid attack.

  CEA 2.9(2020/8)
  SCC 1.0(2020/8)

  2020/8
  @ PATHOLOGY- Bx of rectum (over dental line): poor-differentiated carcinoma with neuroendocrine differentiation
  CT: rectum tumor
  COS: anal tumor 3" biopsy: SCC, PD
  MRI pelvis: anal tumor, no LN. lesion: 2cm. Rt adnexa mass
  PET: anal tumor, no Mets
  CT lung: Neg.
  2020/11/17 S scopy PR of tumor. cT2N0M0
  2020/12/7 MRI: PR. DRE: almost disappear.
  2021/1/4 DRE: hemorrhoid swelling.
  2021/2/1 DRE: hemorrhoid swelling.
  2021/3 MEI: little increase size DRE: suspect PD.

- 2021/3 lung CT neg.
  - General appearance :
    - easy looking , conscious clear , without cardiopulmonary distress
    - Vital signs自動帶入date 體溫(BT) 脈搏(P) 呼吸(R) 血壓(BP) 身高(BH) 體重(BW) BMI
      20210330 35℃ 71次/分 18次/分 140/60mmHg 156.4cm 69.3kg 28.3
    - Vital signs: B.P. 140/60 mmHg , P.R. 71 /min , R.R. 18 /min , B.T. 35 ℃
  - HEENT :
    - Eyes: no anemic conjunctiva, no icteric sclera, normal EOM
    - ENT: no otorrhea, no rhinorrhea, no erythematous mucosa, no injected tonsil, no deformity
  - Neck: supple, freely movable, trachea midline,no deformity, no engorged jugular vein , no palpable thyroid or lymph node enlargement
  - Chest:
    - Inspection: Grossly normal, symmetric and fully expansion, no accessory muscle used, no operation scar, no spider angiomata, no gynecomastia
    - Palpation: no palpable mass or nodule, no subcutaneous emphysema, no tenderness
    - Ausculation: clear breathing sound
  - Heart:
    - Percussion: normal heart size
    - Ausculation: regular heart beat, normal S1, S2, no gallop, no murmur
  - Abdomen:
    - Inspection: in
      - flat contour, symetrically, no scar, no stria, no dilated veins, no rashes
    - Ausculation: normoactive bowel sound, no abdominal bruits
    - palpation: soft, no muscle guarding or rigidity, no local tenderness, no rebounding pain, no palpable mass, no palpable pulsation
    - Percussion: no shifting dulllness, no hepatomegaly, no splenomagaly
  -

Peripheral pulsation :	CA	BA	RA	FA	PA	PTA	DPA
Right side :	++	++	++	++	++	++	++/+++
Left side :	++	++	++	++	++	++	++/+++

  - Genito-anus: no tenderness or palpable mass lesion or rectal shelf, normal external genitalia, normal anal tone, brown stool

CRS CONDITION

- Imaging studies(Sono , CT of abdomen and chest Films):
  - MRI pelvis showed anal cancer post neoadjuvant CCRT, with regression change of tumor size as compare with previous MR on 20201205.
-
  - Lung

手術日期及方法

- 手術日期及方法20210331 Incisional biospy
  20210401 Robot Assisted Low Anterior Resection

住院治療經過

- The patient is admitted for scheduled surgery with robotic-assist LAR due to SCC of anus. During hospitalization, pre-operative survey showed acceptable conditions. She underwent surgery on 2021/4/1 with incisional biopsy. She also received lymphoscintigraphy on the same days which revealed sentinel lymph nodes. After surgery, she went back to ward for post-operative care. Her post-operative recovery as ERAS protocol was satisfactory. We removed the switch on abdomen on 4/7. Under relatively stable condition, she was therefore discharged with OPD follow-up.

合併症

一般檢查

特殊檢查

病理報告

- pending

其它

出院時情況

- 3.改門診治療

出院指示

藥名	劑量	劑量單位	用法	頻次	數量	數量單位	現況	天數	單位
Mag. Oxide tab	2	TAB	PO	TID	42	TAB	TH	7	天
(□)Takepron OD tab 30mg	1	TAB	PO	QDAC	7	TAB	TH	7	天
Ultracet tab	1	TAB	PO	Q6HPRN	12	TAB	TH	3	天
Through tab	1	TAB	PO	QN	7	TAB	TH	7	天

出院計劃

- 門診日期
  - date on 2021/04/13

主治醫師 2222H 林俊余 / 住院醫師 9958C 林強生

正式摘要(電子簽章：000000213950998)

W55 / W55

doctorRank:B
